# Entire Mitral Valve Reconstruction Using Porcine Extracellular Matrix: Adding a Ring Annuloplasty

**DOI:** 10.1007/s13239-024-00727-0

**Published:** 2024-03-19

**Authors:** Johannes H. Jedrzejczyk, Stine Krog, Søren N. Skov, Karen B. Poulsen, Mona Sharghbin, Leila L. Benhassen, Sten L. Nielsen, J. Michael Hasenkam, Marcell J. Tjørnild

**Affiliations:** 1 Department of Cardiothoracic and Vascular Surgery, Aarhus University Hospital, Palle Juul-Jensens Boulevard 99, 8200 Århus, Denmark; 2 Department of Clinical Medicine, Aarhus University Hospital, Århus, Denmark; 3 Department of Surgery, University of the Witwatersrand, Johannesburg, South Africa

**Keywords:** Mitral valve, Mitral valve reconstruction, Mitral valve repair, Mitral valve replacement, 2-Ply small intestinal submucosa extracellular matrix, Annuloplasty ring

## Abstract

**Purpose:**

This study investigated the implications of inserting a flexible annuloplasty ring after reconstructing the entire mitral valve in a porcine model using a previously investigated tube graft design made of 2-ply small intestinal submucosa extracellular matrix (CorMatrix®).

**Methods:**

An acute model with eight 80-kg pigs, each acting as its own control, was used. The entire mitral valve was reconstructed with a 2-ply small intestinal submucosa extracellular matrix tube graft (CorMatrix®). Subsequently, a Simulus® flexible ring was inserted. The characterization was based on mitral annular geometry and valvular dynamics with sonomicrometry and echocardiography.

**Results:**

After adding the ring annuloplasty, the in-plane annular dynamics were more constant throughout the cardiac cycle compared to the reconstruction alone. However, the commissure–commissure distance was statistically significantly decreased [35.0 ± 3.4 mm vs. 27.4 ± 1.9 mm, P < 0.001, diff =  − 7.6 mm, 95% CI, − 9.8 to (−5.4) mm] after ring insertion, changing the physiological annular D-shape into a circular shape which created folds at the coaptation zone resulting in a central regurgitant jet on color Doppler.

**Conclusion:**

We successfully reconstructed the entire mitral valve using 2-ply small intestinal submucosal extracellular matrix (CorMatrix®) combined with a flexible annuloplasty. The annuloplasty reduced the unphysiological systolic widening previously found with this reconstructive technique. However, the Simulus flex ring changed the physiological annular D-shape into a circular shape and hindered a correct unfolding of the leaflets. Thus, we do not recommend a flexible ring in conjunction with this reconstructive technique; further investigations are needed to discover a more suitable remodelling annuloplasty.

## Introduction

Mitral valve repair is considered to have an advantage over mitral valve replacement [1–4]. The operative and long-term mortality is lower for mitral valve repair and is associated with reduced incidence of re-operations and valve complications [1, 2, 5–9]. However, some pathophysiological anatomical alterations can necessitate mitral valve replacement [1, 2, 10, 11].

The mitral valve can be replaced with either a bioprosthetic- or mechanical valve [12, 13]. Patients undergoing bioprosthetic valve replacement can experience valve degeneration, leading to valve tissue calcification, necessitating reoperation after 10–15 years [12–14]. The mechanical valve increases the risk of thrombosis on the valve surface, requiring the patient to receive life-long anticoagulant therapy [6, 13]. These disadvantages and the inferior survival for mitral valve replacement are unsatisfactory and have been the motivation for an improved replacement technique [15–22]. One potential solution is mitral valve reconstruction using a biological scaffold with reconstruction of the entire mitral valvular and subvalvular apparatus, as described for the mitral and tricuspid valves [15–28].

We have previously reconstructed the entire mitral valvular and subvalvular apparatus using 2-ply small intestinal submucosa extracellular matrix, a biological scaffold with inherent re-endothelization properties [16–18]. However, an unphysiological systolic annular widening was found due to a ballooning effect of the mitral annulus [17, 18]. This ballooning effect intuitively increases the stress distribution in the annular transmission zone, compromising the durability [17]. One potential solution to address the unphysiological systolic annular widening is to utilize a ring annuloplasty in combination with our tube graft reconstruction. This approach could address the issue by remodelling and stabilizing the mitral annulus, counteracting unphysiological systolic widening [29].

We hypothesize that reconstructing the entire mitral valve using a modified tube graft made from 2-ply small intestinal submucosa extracellular matrix with an additional insertion of an annuloplasty ring is feasible in an acute porcine study. More specifically, we hypothesize that adding an annuloplasty ring to the reconstruction will stabilize the mitral annulus and thereby counteract the unphysiological systolic annular widening, which was found previously for the reconstruction itself. This study aims to characterize the reconstruction of the entire mitral valve with an additional insertion of a flexible ring annuloplasty. The characterization was based on annular, valvular, and subvalvular dynamics and geometry through sonomicrometry and echocardiography.

## Materials and Methods

### Ethical Statement

The study was approved by the Danish Inspectorate of Animal Experimentation and complied with the National Guidelines for Experimental Animal Research (nr. 2016-15-0201-01132).

### Animals

The study population comprised eight female 6 months old Mixed Yorkshire and Danish Landrace pigs provided by the Aarhus University Experimental Animal Farm, Aarhus, Denmark. All animals were bred under standard laboratory conditions. The porcine species was chosen since the porcine heart is comparable to its human counterpart [30]. Before this study, a pilot study of six animals was performed to design and optimize the surgical technique [18]. The same study population was used in our echocardiographic and sonomicrometric studies describing our new surgical technique [17, 18].

### Reconstruction Material

**Fig. 1:**
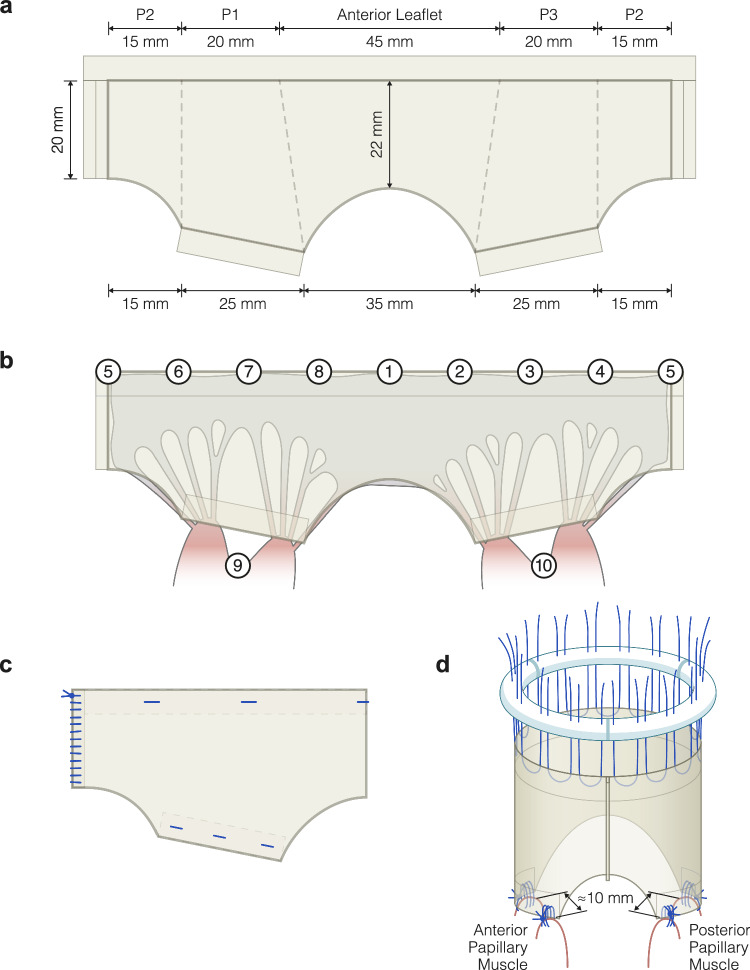
A schematic illustration of the 4.5 × 12.5 cm modified tube graft used for the entire mitral valvular and subvalvular reconstruction. **a** The sheet used for the modified tube graft with measurement values. **b** The sheets resemble the native valve with markings of the sonomicrometry crystals. **c** The sutured tube graft. The dotted boxes indicate double-folded layers. **d** The modified tube with the placement of sutures for the attachment. The attachment of four connective points of the tube graft onto their respective papillary muscle head using the 3-suture loop technique

The overall design of the modified tube graft is illustrated in Fig. 1 and has been described in detail elsewhere [18]. The material was made from a 4 × 12.5 cm sheet of 2-ply small intestinal submucosa extracellular matrix (CorMatrix; Cardiovascular, Inc., Alpharetta, GA), Fig. 1a. The material was double folded at the papillary attachment sites and the annular zone. Figure 1b shows the anatomical differences between the native mitral valve and the modified tube graft and the sonomicrometry crystals’ placement. Figure 1c illustrates the modified tube graft when sutured, and Fig. 1d illustrates the modified tube graft with the annuloplasty ring when placed on the papillary muscles.

### Annuloplasty Ring

We used the Simulus® flexible annuloplasty system, size 31 (Medtronic, Inc., Minneapolis, MN) for the annuloplasty. The ring consists of a barium tungsten and sulfate core with a braided polyester surrounding the core [31]. The specific dimension of the ring has been described in detail elsewhere [15]. The ring size was chosen to establish a down-sizing effect and, thereby, a functional leaflet coaptation [15, 31]. This choice was based on previous studies and native annulus dimensions to ensure compliance with the current clinical practice [1, 15, 31].

### Instrumentation

Mikro-Tip pressure catheters (SPR-350S, Millar Instruments, Houston, TX) were used to measure the left ventricular pressure, left atrial pressure, and aortic pressure. The pressure measurement results for the reconstruction without adding the annuloplasty ring have been described in detail elsewhere [17].

**Fig. 2:**
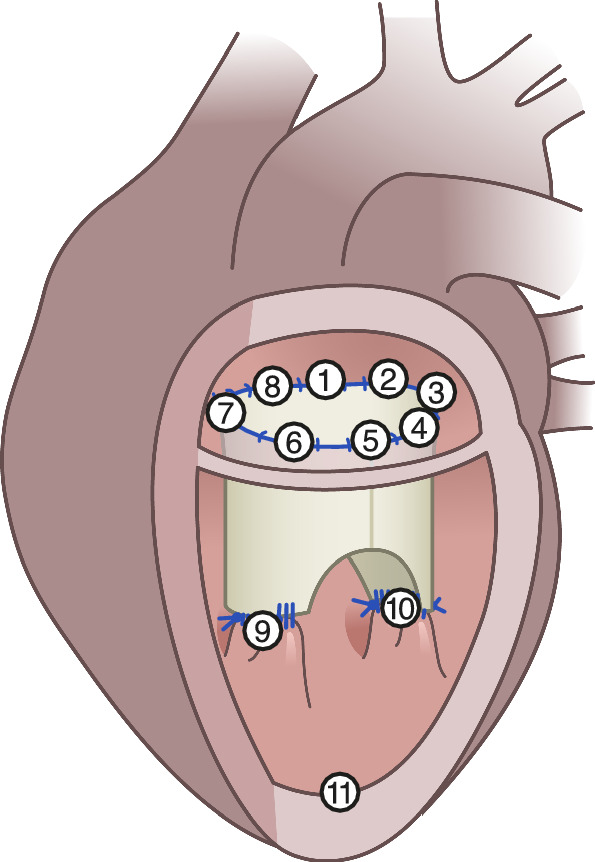
The modified tube graft with implanted sonomicrometry crystals in the mitral annulus and the subvalvular apparatus. The anatomical landmarks defined the crystal position: 1: Centre of trigones; 2: Right trigone; 3: Posterior commissure; 4: P3 scallop; 5: Centre of the posterior annulus (P2 scallop); 6: P1 scallop; 7: Anterior commissure; 8: Left trigone; 9: Anterior papillary muscle; 10: Posterior papillary muscle and 11: left ventricular apex

We used sonomicrometry with 11 piezoelectric sonomicrometry crystals (2 mm) to measure the annular and subvalvular geometry (Sonometrics Corp., London, Canada) [32]. The placement of the sonomicrometry crystals is illustrated in Figs. 1b and 2. The sonomicrometric results for the reconstruction without adding the annuloplasty ring have been described in detail elsewhere [17].

Epicardial echocardiography was performed using a 3-dimensional transoesophageal echocardiographic probe (Vivid E9, GE Vingmed Ultrasound AS, Horten, Norway) to visualize the valve. A color Doppler was used to test for regurgitation. The echocardiographic results for the reconstruction without adding the annuloplasty ring have been described in detail elsewhere [18].

### Study Design

We used an acute 80-kg porcine experimental model, where the pigs acted as their own controls. Echocardiography and invasive pressure measurements were performed after tube graft reconstruction. Subsequently, the sonomicrometry crystals and the annuloplasty ring were inserted, and echocardiography, invasive pressure and sonomicrometry measurements were performed. Finally, the ring annuloplasty was excised through the left atrium on a beating heart, and sonomicrometry measurements with the tube graft reconstruction alone were performed.

### The Surgical Procedure

**Fig. 3:**
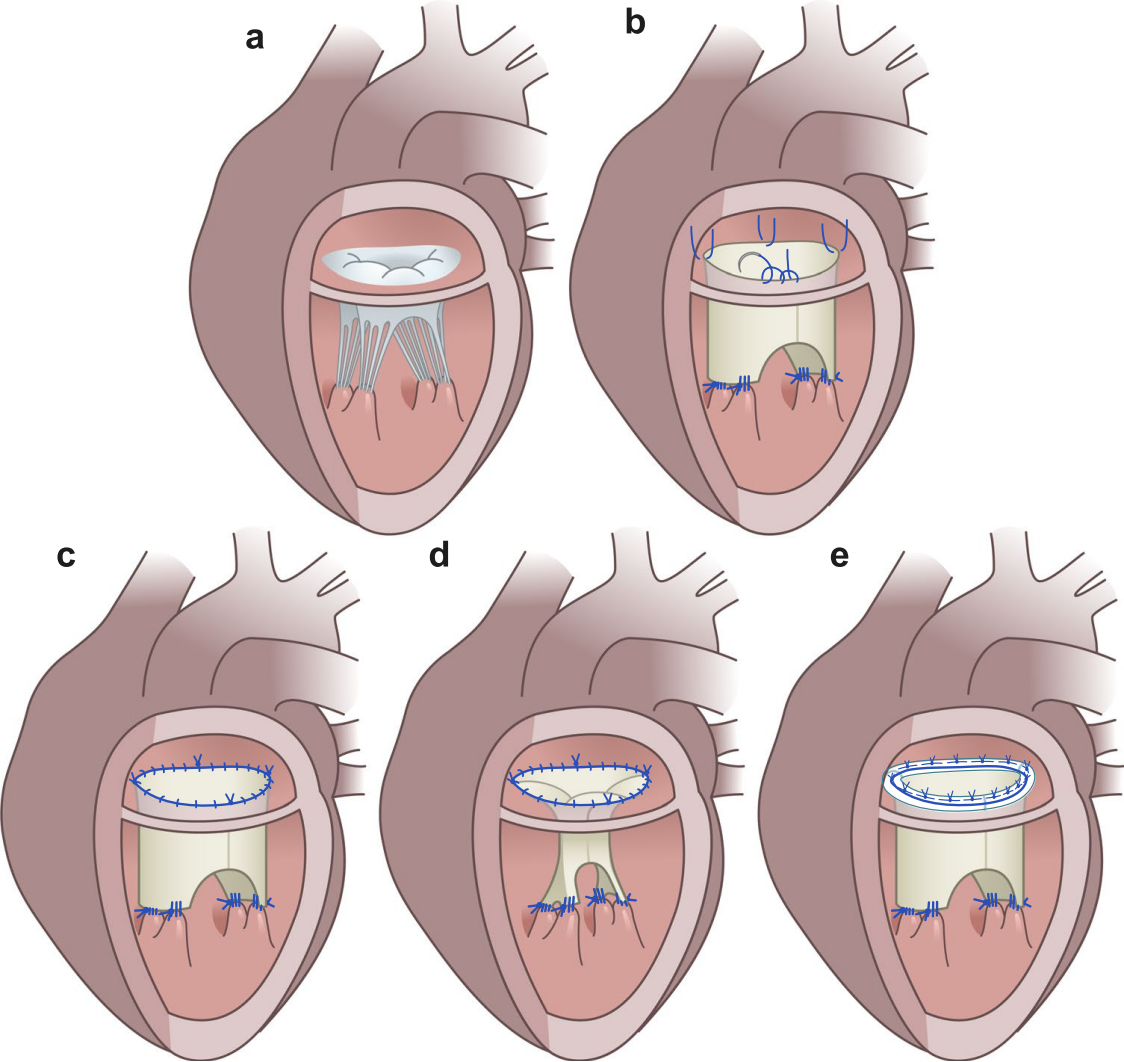
**a** Schematic illustration of the native valve. **b** Insertion of the modified tube graft. **c** Illustration of the reconstruction in diastole. **d** Illustration of the reconstruction in systole. **e** Illustration of the reconstruction with insertion of the annuloplasty ring. The sonomicrometric results for the reconstruction without adding the annuloplasty ring have been previously published [17]

Transportation, medication, handling, surgical technique, and anesthesia of the animals have been described in detail previously [17, 18]. The overall surgical principles of the mitral valvular and subvalvular reconstruction and insertion of the annuloplasty ring can be found in Fig. 3. Figure 3a illustrates the native mitral valve. Figure 3b illustrates the modified tube graft. Figure 3c illustrates the reconstruction in diastole. Figure 3d illustrates the reconstruction in systole. Figure 3e illustrates the reconstruction with the insertion of the annuloplasty ring.

Cardiopulmonary bypass was established, and cardioplegic arrest was initiated. After reconstruction, the animals were weaned off cardiopulmonary bypass and measurements with epicardial echocardiography and hemodynamic data recordings were completed. A second round of cardiopulmonary bypass and cardioplegic arrest were established. Eleven sonomicrometry crystals were placed, eight around the mitral annulus, one on each papillary muscle and one at the apex, Fig. 2. Subsequently, the Simulus® Flex ring was implanted using the parachute technique with 2-0 PremiCron® (B. Braun, Melsungen, Germany), Figs. 1d and 3e. The animals were weaned off cardiopulmonary bypass, and the hemodynamic and sonomicrometry measurements with the annuloplasty ring inserted were performed.

The third and final round of cardiopulmonary bypass was established. The ring annuloplasty was excised through the left atrium on a beating heart, and the atrium was closed. The animals were weaned off cardiopulmonary bypass, and sonomicrometry measurements without the ring were performed.

### Data acquisition

The data acquisition has been described previously [17, 18]. We recorded hemodynamic and sonomicrometric data for ten consecutive heart cycles. We used the time derivate of the left ventricular pressure dLVP/dt to synchronize the analogue hemodynamic and sonomicrometric signals.

Throughout the cardiac cycle, four cyclic time points were defined; start systole as the dLVP/dt maximum, mid-systole as the midpoint between dLVP/dt maximum and minimum, end-systole as the dLVP/dt minimum, mid-diastole as the midpoint between end-systole and the following dLVP/dt maximum and, finally, end-diastole presented as the R-peak on the electrocardiography.

We used virtual instrumentation software (LabVIEW 15.0, National Instruments, Austin, TX) to record all analogue data signals and a pressure control unit (PCU-2000, Millar Instruments) to amplify the signals from the Mikro-Tip pressure catheters. We analyzed all data offline and defined two mean pressure differences: The mitral valve between end-systole and end-diastole and the aortic valve between start-systole and end-systole. The electrocardiographic data were monitored and stored in a CardioMed system (Model 4008, CardioMed A/S, Oslo, Norway). A multi-dimensional scaling technique was used for post-processing, depicting each crystal in a Cartesian coordinate system (SonoSOFT and SonoXYZ, Sonometrics Corp).

The mitral annular circumference was calculated using the summarized distances between the eight annular crystals. The adjacent triangular areas were used to calculate the mitral annular area. The septal lateral distance was calculated directly between Crystal 1 and 5, the commissure–commissure distance was calculated between Crystal 3 and 7, and the inter-papillary muscle distance from Crystal 9 to 10, Fig. 2. Crystal 7 and 3 were used as landmarks for dividing an anterior segment and a posterior segment of the mitral annulus. The posterior annular segment spanned from Crystal 3 to 7, and the anterior annular segment spanned from Crystal 7 to 3, Fig. 2.

A least-square plane was fitted from the annular crystals. The individual annular-crystal height to the least-square plane was calculated as an indicator of the mitral saddle shape. The sum of the highest crystal-plane distance on each side of a least-square plane was used to calculate the annular height. The annular height-to-commissural width ratio was calculated as the ratio between annular height and commissure–commissure distance.

### Statistical Analysis

The sample size was based on previous studies with similar testing setups and was cautiously estimated using $n=\frac{2{({Z}_{\alpha }+{Z}_{1-\beta })}^{2}{\sigma }^{2}}{{\Delta }^{2}}$ [30]. ${Z}_{\alpha }$ is a constant of 1.96 for the two-sided effect; ${Z}_{1-\beta }$ is a constant of 0.8416 for a power of 80%; σ is the estimated standard deviation, in our study 10% and ∆ is the estimated difference in the effect of the two interventions, in our study 15% [33]. We used a sample size of n = 8, as the estimated sample size was a minimum of n = 7.

All data were analyzed using STATA version 13 (StataCorp LP, College Station, TX) and reported as a mean ± standard deviation with a significance level of P < 0.05. The statistical model was applied and developed with support from Aarhus University (BIAS, University of Aarhus, Aarhus, Denmark).

Hemodynamic and sonomicrometric parameters at two time point measurements, defined as before and after ring insertion, were compared using a mixed model with repeated measurement analysis, with time as a fixed effect and pig and time within pig as random effects.

Echocardiographic parameters were compared at the two time points using a mixed-model, repeated-measurement analysis with time as a fixed effect and pig as a random effect.

Both models allowed for different residual variations at the two measurement time points. Model diagnostics were performed by inspecting residuals plotted against the fitted values and normal quantile plots of the residuals. None of these assessments gave any reason to doubt the validity of the analysis. The two-measurement time points were compared using post hoc Wald Z-tests.

One signal measurement round from Crystal 2 and the mitral valve pressure difference from one signal measurement round with the ring were excluded due to poor signal.

## Results

### Hemodynamic Results

The hemodynamic parameters and pressure differences before and after ring insertion are presented in Table 1. There was a statistically significant increase in the peak left atrial pressure [20 ± 7 mmHg vs. 27 ± 5 mmHg, P < 0.005, diff = 6.8 mmHg, 95% CI (confidence interval) 2.1–11.6 mmHg] before and after ring insertion.

**Table 1:** Haemodynamic parameters

Parameter	Reconstruction sonomicrometry	Reconstruction annuloplasty ring	*P*-value	Diff	95% CI
	(n = 8)	(n = 8)			
Heart rate (min^−1^)	101 ± 17	93 ± 11	0.099	− 8	− 15.8 to 1.4
Ejection fraction (%)	67 ± 7	67 ± 9	0.76	0	− 4.1 to 5.6
Catheter-based pressure measurements					
Peak LVP (mmHg)	86 ± 13	82 ± 16	0.62	− 4	− 17.6 to 10.5
Peak of LVP change (dLVP/dt_Max_) (mmHg/s)	1666 ± 583	1342 ± 383	0.20	− 324	− 816 to 167
Peak LAP (mmHg)	20 ± 7	27 ± 5	0.005*	6.8	2.1 to 11.6
Mitral valve pressure difference (mean) (mmHg)	8.3 ± 3.4	9.7 ± 4.1	0.054	1.4	− 0.2 to 2.5
Aortic valve pressure difference (mean) (mmHg)	9.7 ± 5.5	9.5 ± 4.7	0.89	− 0.2	− 3.5 to 3.1

Hemodynamic parameters after reconstruction with sonomicrometry before and after ring insertion. Values are presented as mean ± standard deviation and as a comparison between two time points. The hemodynamic results for the reconstruction without adding the annuloplasty ring have been previously published [17]

*Diff* difference between the two time points, *dLVP/dt* time derivate of the left ventricular pressure, *LAP* left atrial pressure, *LVP* left ventricular pressure

*P < 0.05

### Sonomicrometry

**Fig. 4:**
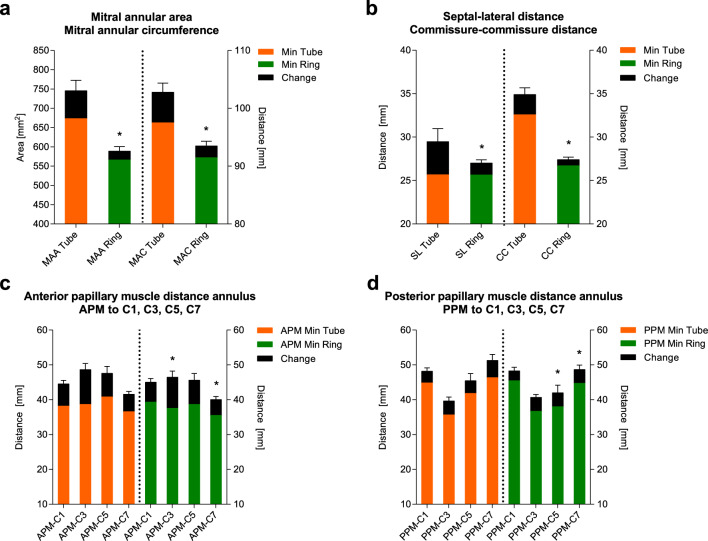
The maximum, minimum and change values with standard deviation before and after ring insertion for the **a** mitral annular area and mitral annular circumference, **b** septal–lateral distance and commissure–commissure distance, **c** anterior papillary muscle and **d** posterior papillary muscle distances to the mitral annulus. Error bars show standard deviations. *P < 0.05 for the maximum value between the two time points. The sonomicrometric results for the reconstruction without adding the annuloplasty ring have been previously published [17]

The maximum, minimum and change values for the mitral annular area, mitral annular circumference, septal–lateral and commissure–commissure distance are presented before and after ring insertion in Fig. 4a, b. There was a statistically significant decrease in the mitral annular area [MAA_Max_ 755 ± 100 mm^2^ vs. 589 ± 77 mm^2^, P < 0.001, diff =  − 166 mm^2^, 95% CI − 220 to (−111) mm^2^] and the mitral annular circumference [MAC_Max_ 103.3 ± 7.3 mm vs. 93.5 ± 5.4 mm, P < 0.001, diff =  − 9.8 mm, 95% CI − 13.8 to (−5.7) mm] before and after ring insertion. A similar statistically significant decrease was found for the septal–lateral [SL_Max_ 29.7 ± 1.7 mm vs. 27.0 ± 3.0 mm, P = 0.016, diff =  − 2.7 mm, 95% CI − 4.9 to (−0.5) mm] and commissure–commissure distance [CC_Max_ 35.0 ± 3.4 mm vs. 27.4 ± 1.9 mm, P < 0.001, diff =  − 7.6 mm, 95% CI − 9.8 to (−5.4) mm] before and after ring insertion.

The maximum, minimum and change values for the distances from the anterior and posterior papillary muscle to the annular plane at the A2 scallop, posterior commissure, P2 scallop and anterior commissure are also presented before and after ring insertion in Fig. 4c, d. For the anterior papillary muscle, there was a statistically significant decrease at the posterior commissure [APM-C3_Max_ 48.7 ± 3.4 mm vs. 46.5 ± 3.2 mm, P = 0.003, diff =  − 2.2 mm, 95% CI − 3.7 to (−0.7) mm] and to the anterior commissure [APM-C7_Max_ 41.5 ± 3.5 mm vs. 40.1 ± 4.2 mm, P = 0.002, diff =  − 1.4 mm, 95% CI − 2.4 to (−0.5) mm]. For the posterior papillary muscle, there was a statistically significant decrease at the anterior commissure [PPM-C7_Max_ 51.4 ± 3.2 mm vs. 48.7 ± 4.2 mm, P = 0.004, diff =  − 2.7 mm, 95% CI − 4.6 to (−0.9) mm] and to the P2 scallop [PPM-C5_Max_ 45.3 ± 5.2 mm vs. 42.0 ± 4.2 mm, P < 0.001, diff =  − 3.3 mm, 95% CI − 5.3 to (−1.4) mm], before and after ring insertion.

**Fig. 5:**
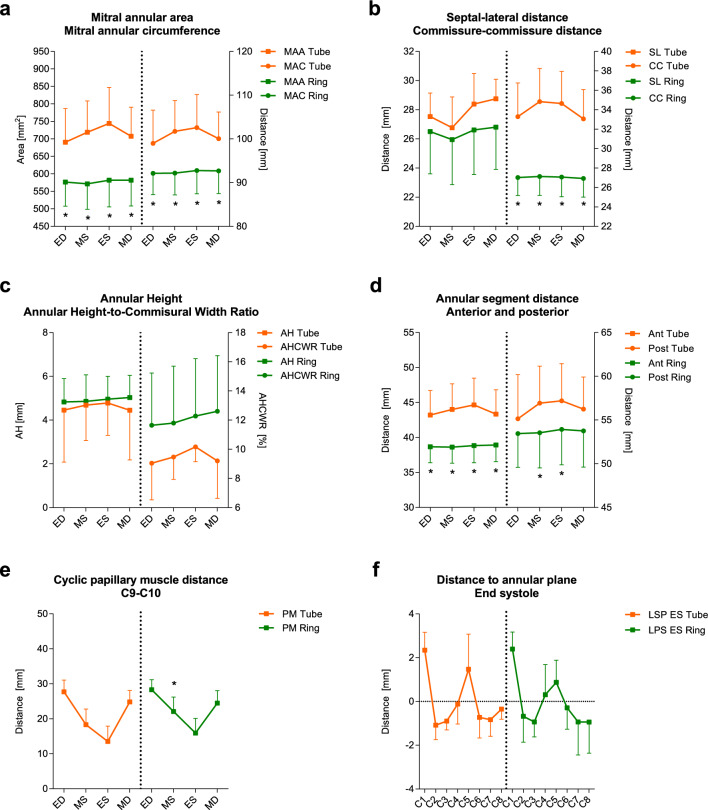
**a** The cyclic change for the mitral annular area and mitral annular circumference before and after ring insertion. **b** The cyclic change for the septal–lateral distance and the commissure–commissure distance before and after ring insertion. **c** The cyclic change for the annular height and the annular height-to-commissural width ratio before and after ring insertion. **d** The cyclic changes of the anterior and posterior annular segmental distances before and after ring insertion. **e** The cyclic changes of the interpapillary muscle distance. **f** The distance from the annular crystals to the least square plane before and after ring insertion as an expression of the mitral annular saddle shape. The error bars show standard deviations. *P < 0.05 between the two time points. The sonomicrometric results for the reconstruction without adding the annuloplasty ring have been previously published [17]

The cyclic change for the mitral annular area, mitral annular circumference, septal–lateral and commissure–commissure distance, annular height, annular height-to-commissural width ratio, anterior and posterior annular segmental distances and interpapillary muscle distance are presented before and after ring insertion in Fig. 5a–e. Throughout the cycle, there was a statistically significant decrease in the mitral annular area, mitral annular circumference, commissure–commissure distance and anterior annular segmental distance. These changes were at a more constant level compared with the tube graft reconstruction without the annuloplasty ring.

As an expression of the mitral annular saddle shape, distances of the annular crystals to least square plane are presented before and after ring insertion in Fig. 5f.

**Fig. 6:**
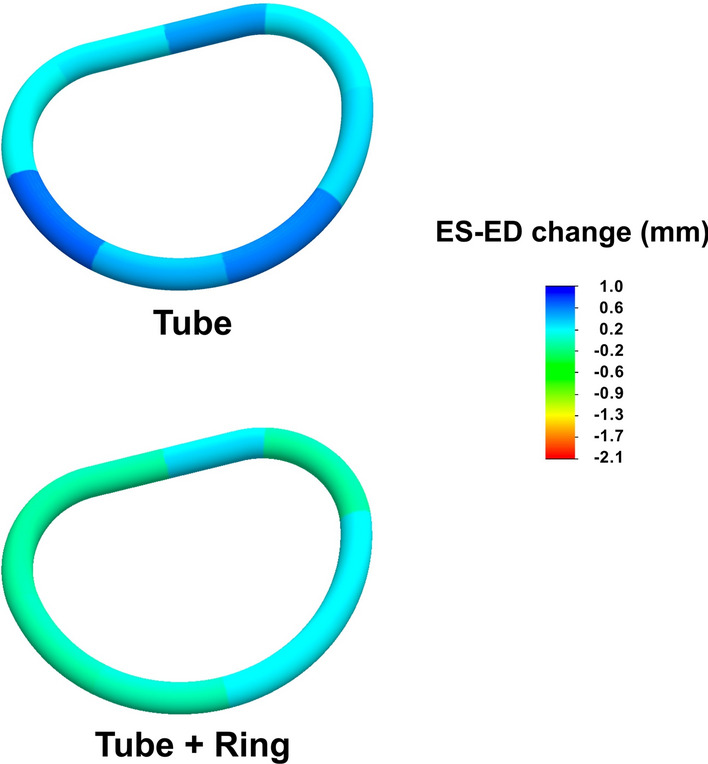
A scaled color legend illustrating the annular segmental circumferential changes from end-diastole to end-systole before and after ring insertion. The blue color represents a systolic expansion of the circumference, and the red represents a systolic compression of the circumference. The sonomicrometric results for the reconstruction without adding the annuloplasty ring have been previously published [17]

As an expression of the regional systolic circumferential changes, the annular segmental circumferential changes from end-diastole to end-systole are illustrated in Fig. 6, with a scaled color legend before and after ring insertion. Blue represents a systolic expansion of the circumference, and red represents a systolic compression of the circumference.

### Echocardiographic Results

The echocardiographic parameters before and after ring insertion are listed in Table 2. The echocardiographic assessment revealed fully functional valves without any echocardiographic signs of obstruction due to systolic anterior motion (SAM). A 5-chamber color Doppler analysis after ring insertion revealed a central regurgitant jet with a vena contracta of 3 mm that was not present before ring insertion.

**Table 2:** Echocardiographic parameters in an apical five chamber view

Parameter	Reconstruction sonomicrometry	Reconstruction annuloplasty ring	P-values	Diff	95% CI
	(n = 8)	(n = 8)			
Annulus diameter (SL) (mm)					
Systole	21.8 ± 1.3	19.4 ± 0.5	< 0.001*	− 2.4	− 3.3 to − 1.4
Diastole	23.9 ± 2.3	19.8 ± 0.5	< 0.001*	− 4.1	− 5.8 to − 2.5
Cyclic change	2.1 ± 1.8	0.4 ± 0.5	0.008*	− 1.7	− 3.1 to − 0.4
Systole anterior leaflet part	10.8 ± 0.9	10.3 ± 0.7	0.14	− 0.5	− 1.2 to 0.2
Systole posterior leaflet part	10.8 ± 1.7	9.4 ± 0.9	0.029*	− 1.5	− 2.6 to − 0.8
Tenting area (mm^2^)	45.0 ± 14.1	67.5 ± 13.9	0.011*	22.5	5.2 to 39.8
Anterior leaflet part	25.0 ± 9.3	35.0 ± 9.3	0.009*	10	2.5 to − 17.5
Posterior leaflet part	22.5 ± 7.1	32.5 ± 7.1	0.035*	10	0.7 to 19.3
Billowing height (mm)					
Anterior leaflet	1.9 ± 1.6	4.0 ± 0.8	< 0.001*	2.1	1.0 to 3.2
Posterior leaflet	2.9 ± 1.5	3.8 ± 0.8	0.072	0.9	0.1 to 1.8
Tenting height (mm)	5.8 ± 1.6	6.8 ± 1.2	0.11	1.0	− 0.2 to 2.2
Coaptation length (mm)	7.9 ± 1.0	9.1 ± 0.8	0.048*	1.2	0.0 to 2.5
Leaflet length (mm)					
Anterior leaflet	23.1 ± 1.7	23.4 ± 1.7	0.77	0.3	− 1.4 to 1.9
Posterior leaflet	18.9 ± 1.2	18.8 ± 1.2	0.84	− 0.1	− 1.3 to 1.0

Echocardiographic parameters in an apical five-chamber view with sonomicrometry before and after ring insertion. Values are presented as mean ± standard deviation and as a comparison between two time points. *Billowing height* is defined as positive in the apical direction. The echocardiographic results for the reconstruction without adding the annuloplasty ring have previously been published [18]

*SL* septal–lateral

^*^P < 0.05

## Discussion

This study is the first to describe the effects of adding a ring annuloplasty to an entire mitral valve and subvalvular reconstruction in an acute porcine model using a modified tube graft from a sheet of 2-ply small intestinal submucosa extracellular matrix. Previous studies have demonstrated that reconstruction using an extracellular matrix resulted in an unphysiological systolic annular widening due to a ballooning effect, possibly increasing the stress distribution of the material in the annular zone [17, 18]. A flexible annuloplasty ring is often added in mitral valve reconstructive surgery to restore annular geometry in a dilated annulus and to enhance leaflet coaptation [1, 34]. Inserting a ring annuloplasty might counteract this unphysiological systolic widening by remodeling and stabilizing the mitral annulus [17, 29]. We used a flexible ring annuloplasty because they are expected to preserve the physiological cyclic changes of the annulus [29, 35–37]. The implications of adding a ring annuloplasty were successfully characterized through mitral annular geometry and valvular dynamics with sonomicrometry and echocardiography.

The valve proved functional following the reconstruction, both with and without the ring. Adding the ring annuloplasty stabilized the mitral annulus and thereby counteracted the unphysiological systolic annular widening found after reconstruction without the ring annuloplasty. However, on echocardiography, we found excessive leaflet material at the coaptation zone, which resulted in mild to moderate regurgitation with the ring annuloplasty in situ.

Adding a ring annuloplasty resulted in a general downsizing of the mitral annulus, as seen for the mitral annular area and the mitral annular circumference, in line with the general clinical practice [1, 34]. This general downsizing did not result in any stenosis since the mitral valve pressure difference was comparable before and after adding the ring annuloplasty. Consequently, we assess this downsizing to be of less clinical importance. However, the downsizing did not benefit the mitral reconstruction, which indicated that choosing a normo-sized ring could be advisable. Nevertheless, the downsizing could benefit patients with a pathophysiological dilation of the mitral annulus. Such conditions should be considered when performing heart valve surgery, as they can create an increased cardiac workload causing pathophysiological alterations of the heart.

The septal–lateral distance was also downsized after adding the ring annuloplasty, which in line with the general clinical practice, resulted in an increased coaptation length. Since the coaptation length with and without the ring annuloplasty is within the recommended 8 to 10 mm range, the clinical importance must be considered negligible [38]. In general, we see these findings for the mitral annular area, mitral annular circumference, and septal–lateral distance of less clinical importance as we control the measurements of the designed valve. With a designed valve, we do not need to apply the general clinical practice, since we can increase the coaptation length by increasing the leaflet length of the designed valve. Thus, we can keep a physiological-sized mitral annulus and still have a satisfactory coaptation.

Downsizing the commissure–commissure distance after adding the annuloplasty is more critical. It was downsized to a comparable degree to the downsized septal–lateral distance. Consequently, the physiological annular D-shape changed its configuration into a circular shape. These findings confirm the observations of previous studies using this specific annuloplasty ring [15, 31]. The change to a circular mitral annulus configuration has a clinical impact. The physiological annular D-shape and physiological systolic expansion of the anterior annular segment result in unfolded leaflet coaptation. Consequentially, the circular annular shape created folds of the leaflets at the coaptation zone. This was indicated on the echocardiography with excessive leaflet material not found for the reconstruction without the ring annuloplasty. The effect was mild to moderate mitral valve regurgitation with the ring annuloplasty. For this reason, we cannot recommend this specific annuloplasty ring with our reconstruction technique. A more rigid ring wither higher remodelling capabilities, and thus greater ability to maintain the septal–lateral and commissure–commissure dimensions [29, 37] may be more suitable with our tube graft design. Thus, further investigations are needed to evaluate if other ring annuloplasties will result in a more physiological annular D-shape when combined with our reconstruction technique.

The ring was proven to keep the physiological saddle configuration, thus confirming it to be flexible out of plane and able to follow the mitral annulus. Regardless, some patients with general mitral annular dilation could benefit further from a ring with additional remodeling properties. A semi-rigid or rigid ring will allow for less change in the septal–lateral- and commissural–commissural dimensions, thereby possessing greater remodeling properties [35, 37]. Furthermore, these ring types are designed with a saddle shape to ensure valve competency by preserving the annular height-to-commissural width ratio that tends to be reduced with annular dilatation [37]. Implantation of the annuloplasty ring significantly reduced the maximum distance between the posterior papillary muscle to the P2 segment of the posterior mitral valve leaflet. No significant change in the maximum distance between the anterior papillary muscle to the A2 segment of the anterior mitral valve leaflet was found. These findings indicate the direct implications of the annuloplasty on the subvalvular geometry when combined with our tube graft design. These geometrical changes may alleviate the previously described weak point at the subvalvular attachment site of the posterior segment in the tube graft [16, 17] and reduce the risk of patch failure. However, further chronic porcine studies are needed to evaluate this.

An alternative to inserting an annuloplasty ring could be to design the tube graft with the desired circumference and use the annular sutures for the modified tube graft to downsize the pathophysiological dilation, leaving the annuloplasty ring redundant. Findings by Arbona M. et al. have suggested that SIS-ECM will fail when placed into contact with a foreign material such as a valve prosthesis or mitral annuloplasty ring [39]. Thus, further enhancing the tube graft design may be superior to the incorporation of an annuloplasty ring. More to this point, it might be worth evaluating whether a resorbable suture would be advantageous for this suture annuloplasty since no foreign material would be left over time, thus facilitating the repopulation and degeneration of the bioscaffold. For the repopulation of cells to be feasible, a sufficient amount of tissue stress may be required, and it remains unclear whether the annuloplasty ring will decrease or affect the patch’s cell growth capacity in any way. Thus, further chronic studies are warranted to assess this.

It will require a chronic study to determine the mid- and long-term effects of a ring annuloplasty. Our study did not exclusively produce beneficial results, and further investigations are needed to optimize this reconstructive technique further. As the material used was 2-ply small intestinal submucosa extracellular matrix with re-endothelization and tissue remodeling properties, further investigations are needed to conclude whether the reconstruction is durable in the mid and long-term with the ring annuloplasty.

### Limitations

This study has some inherent limitations that must be considered when interpreting the results. First, all animals included in this study were healthy and without cardiac pathology or pathophysiological deformities of the heart, otherwise seen in humans undergoing mitral valve replacement surgery. Second, all data were collected directly after complex cardiac surgery with three rounds of extracorporeal circulation and two rounds of cardioplegic arrest. Thus, the interpretation in a clinical setting must be performed cautiously. The study design could have been optimized by randomizing the order of annuloplasty implantation and mitral valve reconstruction procedure. Third, the mitral valve reconstruction was performed using a standardized 80-kg porcine model with a one-sized tube graft. Individual anatomic variations can be observed using a biological model, which might affect the results. Finally, all interventions were performed in an acute animal model with a limited number of animals included. Therefore, this study cannot provide information on mid- to long-term effects such as histological or biomechanical parameters. The study design could have been improved by delaying animal sacrifice 48 h post-implantation to attain initial histological data. However, all experimental animals in this study were euthanized shortly after the final data collection.

## Conclusion

We successfully reconstructed the entire mitral valve using a sheet of 2-ply small intestinal submucosal extracellular matrix in conjunction with a ring annuloplasty. Inserting a flexible annuloplasty ring reduced the unphysiological systolic widening previously found with this reconstructive technique. However, it also resulted in a configurational change from a natural D-shaped annulus to a circular-shaped annulus. Thus, the Simulus® flex ring hindered a correct unfolding of the leaflets resulting in central regurgitation. Adding an annuloplasty ring to our tube graft design decreased the distance from the posterior papillary muscle to the annulus, alleviating stress from the posterior segment of the tube graft and possibly reducing the risk of patch failure. Further investigations are needed to identify a more suitable ring annuloplasty and to evaluate this reconstructive technique’s mid and long-term effects with and without the ring annuloplasty.
